# Dataset from the uniaxial tensile testing of human curly hair fibers under different conditions

**DOI:** 10.1016/j.dib.2023.109943

**Published:** 2023-12-12

**Authors:** Lebogang Mathebela, Rudzani Sigwadi, Tetelo Lekwana, Harry Ngwangwa, Thanyani Pandelani, Fulufhelo Nemavhola

**Affiliations:** a Unisa Biomechanics Research Lab, Department of Mechanical Engineering, School of Engineering, College of Science Engineering and Technology, University of South Africa, Pretoria, 0001, South Africa; b Department of Chemical Engineering, School of Engineering, College of Science Engineering and Technology, University of South Africa, Pretoria, 0001, South Africa; c Department of Mechanical Engineering, Faculty of Engineering and the Built Environment, Durban University of Technology, Durban, 4000, South Africa

**Keywords:** Curly hair, Specimen, Strain rate, Temperature, Female

## Abstract

Individual human hair fibers exhibiting a curly morphology were procured from a female donor within her early thirties (30s). The selected hair fibers donor had refrained from undergoing any form of chemical treatment, including dyeing, relaxing, and bleaching, for a minimum period of six (6) months prior to specimen collection. The isolated single fibers were subjected to uniaxial tensile testing at various strain rates (10^0^.s^−1^,10^−2^. s^−1^ 10^−3^. & 10^−4.^s^−1^). Furthermore, the specimens underwent testing under dry conditions at a temperature of 25°C, as well as full immersion in a saline solution at both 25°C and 35°C. The ensuing mechanical attributes, encompassing engineering was analyzed following the tensile testing.

Specifications Table

**Table:** 

Subject	Material mechanics
Specific subject area	Material characterisation
Data format	Raw, Analyzed, Filtered
Type of data	Table, Graph, Figure
Data collection	The data were collected from the African female individual in her early 30s to avoid the variation in mechanical properties influenced by age, gender, and ethnicity. The specimens were collected from the nape or occipital regions of the scalp as it is not too exposed to sunlight or other external environmental attacks. There was no cosmetic treatment done on the hair for the last six months, but only daily washing in water and drying was done on the hair prior to sample harvest. Curly hair specimens (N=116) were tensile tested with the Ustretch 44N load cell. The fibers were further divided into four groups of n = 10 for every strain-rate 100.s-1,10-2 and 10-3. s-1, and 10-4 under different conditions (Dry and Wet) and temperatures (25°C and 35°C)) at the relative humidity 65% except for the 10-4 dry tests where the test numbers were n= 6. The paper frame technique was used for the clamping of single hair fibers to the tensile machine. The lags on the specimen were removed by hand when gluing the specimen onto the paper. The fabric glue was used to glue the specimen onto the paper tabs. The force and displacement results from the tensile machine were extracted from the tensile machine and further processed into stress-strain curve for curly hair fibers.
Data source location	Place: University of South Africa, Biomechanics Research Lab City/Town/Region: Florida, Gauteng Country: South Africa Latitude and longitude for collected samples/data: -26.161255, 27.900602
Data accessibility	Repository name: Mendeley Data Data identification number: doi: 10.17632/ycp6n7ym24.2 Direct URL to data: https://data.mendeley.com/datasets/ycp6n7ym24/2 Instructions for accessing these data

## 1 Value of the Data

• The data will be useful in understanding the hair mechanics of single curly fiber
• The data presents a full presentation of uniaxial mechanical test for single human curly hair fiber and this data is scarce in the literature especially for curly hair of African descent
• The data presented demonstrates the mechanical and structural anisotropy of single curly hair fiber of dry @ 25°C and in saline @ 25°C and 350C under different strain rates
• First report to characterize the tensile properties of single curly fiber in dry condition 250C and wet condition @ 25°C and 35°C under 100.s-1, 10-2. s-1, 10-3. s-1 & 10-4. s-1 strain rate
• The presented mechanical data can utilize in constitutive modelling of single curly fiber
• This data is useful in the development of computational models to study the detailed behaviour of single human curly fiber in various conditions.

## 2 Background

Human hair is a complicated fibrous heterogeneous material highly affected by numerous factors such as hygiene, ethnicity, environment, and chemical treatment. Curly hair fibers have been recognized as displaying heightened susceptibility to mechanical strain in comparison to straight and wavy fibers [1]. This particular hair type exhibits a non-uniform cross-sectional configuration characterized by twists and bends, resulting in constricted cross-sectional areas along the filament length. Consequently, these areas serve as points of vulnerability, as the application of excessive force can engender localized high-stress regions over the diminutive cross-sectional regions of the filament [1]. Such hair fibers are prevalent within the African population, yet have garnered limited scientific attention. Within the realm of cosmetic literature, investigations pertaining to the potency and flexibility of hair fibers have primarily revolved around mechanical and chemical treatments. It follows that the cosmetic industry could delve further into comprehending the intricate behavior of curly hair, thereby facilitating the development of specialized treatments tailored exclusively for this hair type. This study centers upon evaluating the influence of strain rates on individual curly fibers under both dry and wet conditions, across varying temperatures.

## 3 Data Description

The shared data describe the mechanical properties of single curly from female individual at her early 30s. Uniaxial mechanical test were conducted under varying strain rates (10^0^.s^−1^, 10^−2^. s^−1^, 10^−3^. s^−1^ & 10^−4^. s^−1^) at different temperatures and wetness (25°C dry condition, 25^°^C wet condition and 35°C wet conditions) as shown in Table 1.

**Table 1:** Test matrix for 25^°^C dry condition, 25^°^C wet condition and 35°C wet condition.

Strain Rate	Temperature	Condition	Number of tests
10^0^.s^−1^	25^°^C	Dry	10
		Wet	10
	35^°^C	Wet	10
10^−2^. s^−1^	25^°^C	Dry	10
		Wet	10
	35^°^C	Wet	10
10^−3^. s^−1^	25^°^C	Dry	10
		Wet	10
	35^°^C	Wet	10
10^−4^. s^−1^	25^°^C	Dry	6
		Wet	10
	35^°^C	Wet	10
Total			**116**

**Fig. 1:**
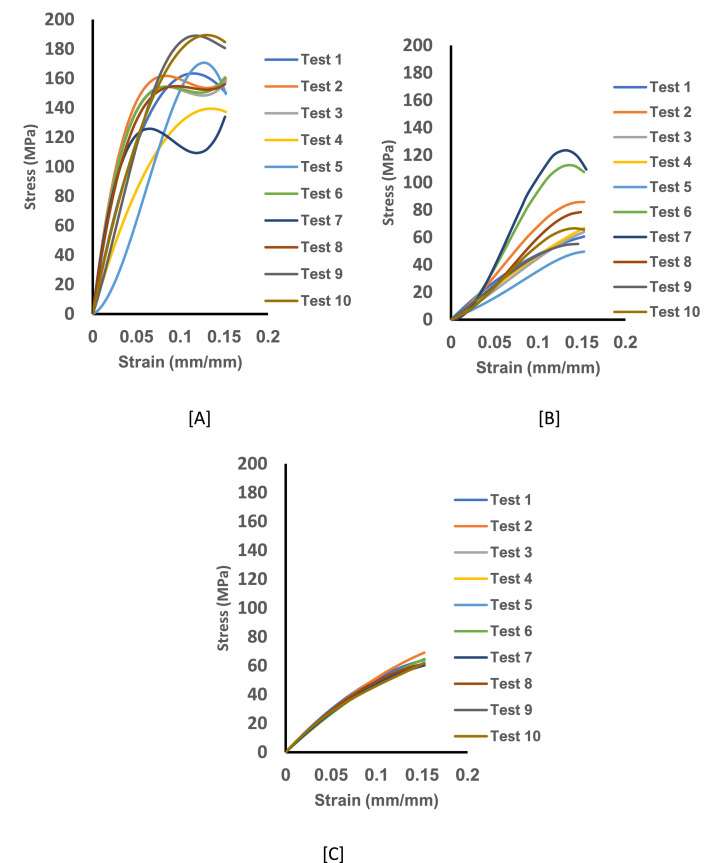
The experimental stress-strain curve data for untreated curly hair showing Test 1-10 from the axial load for specimen pulled for 10^0^.s^−1^. [A] The stress-strain curve for specimen at 25 ℃ and dry condition. [B] The stress-strain curve for specimen at 25 ℃ and wet condition and [C] The stress-strain curve for specimen at 35 ℃ and wet condition.

**Fig. 2:**
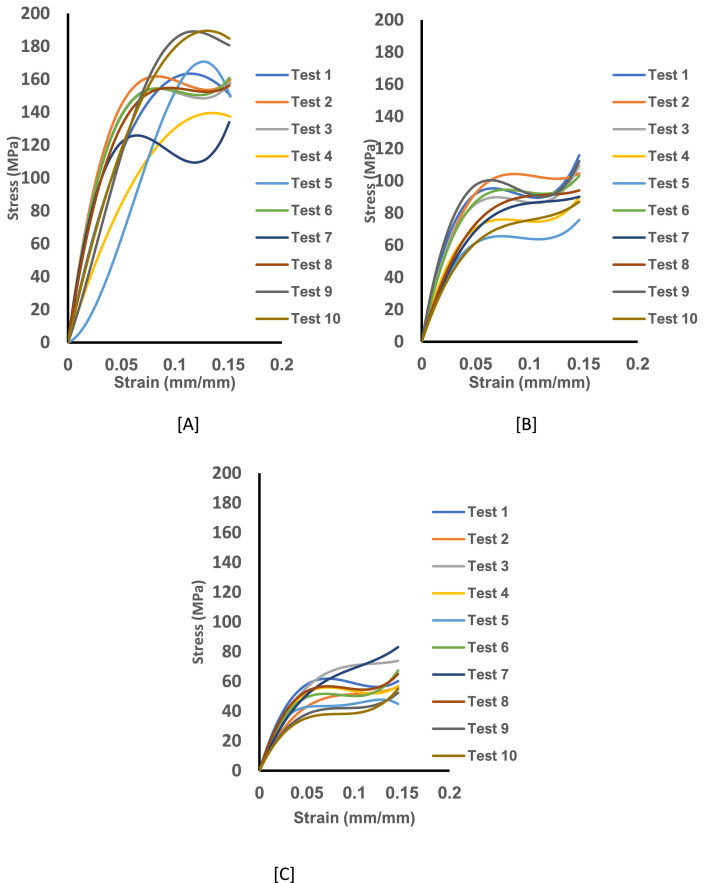
The experimental stress-strain curve data for untreated curly hair showing Tests 1-10 from the axial load for specimen pulled for 10^−2^.s^−1^. [A] The stress-strain curve for specimen at 25 ℃ and dry condition. [B] The stress-strain curve for specimen at 25 ℃ and wet condition and [C] The stress-strain curve for specimen at 35 ℃ and wet condition.

**Fig. 3:**
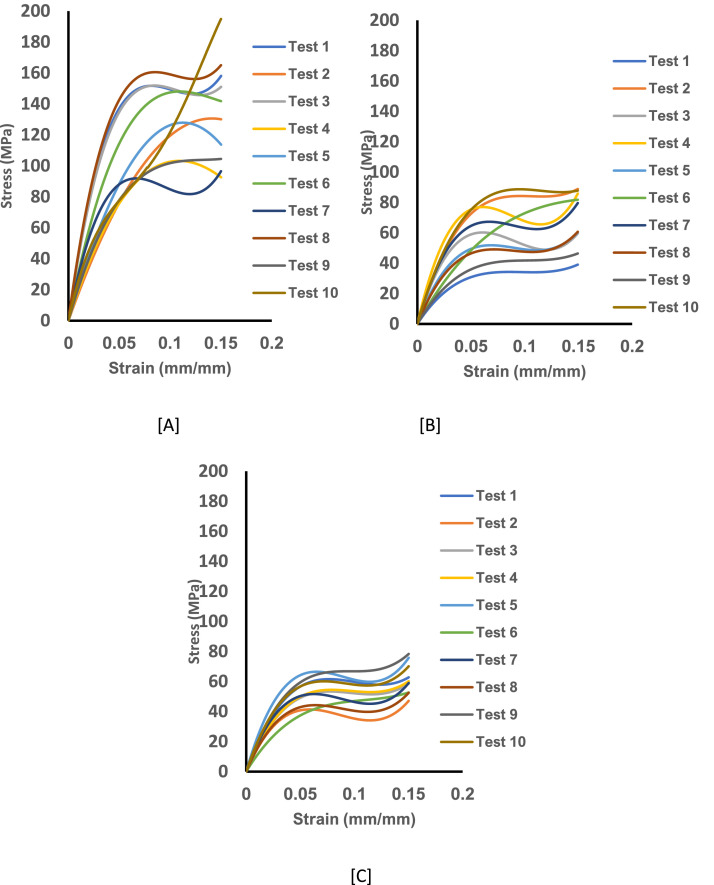
The experimental stress-strain curve data for untreated curly hair showing Tests 1-10 from the axial load for specimen pulled for 10^−3^.s^−1^. [A] The stress-strain curve for specimen at 25 ℃ and dry condition. [B] The stress-strain curve for specimen at 25 ℃ and wet condition and [C] The stress-strain curve for specimen at 35 ℃ and wet condition.

**Fig. 4:**
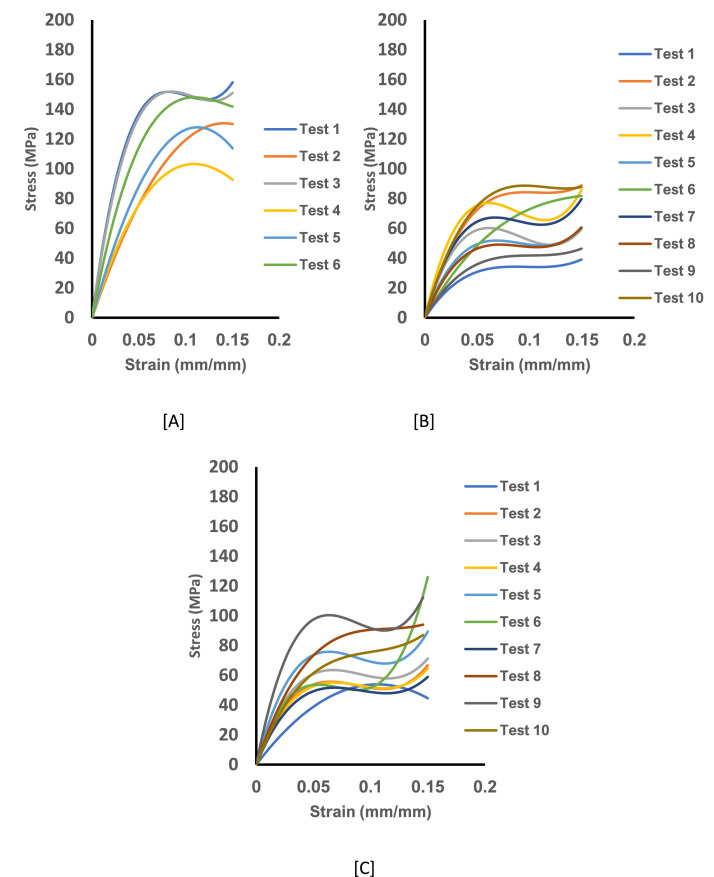
The experimental stress-strain curve data for untreated curly hair showing Tests 1-6 from the axial load for specimen pulled for 10^−4^.s^−1^. [A] The stress-strain curve for specimen at 25 ℃ and dry condition. [B] The stress-strain curve for specimen at 25 ℃ and wet condition and [C] The stress-strain curve for specimen at 35 ℃ and wet condition.

In the published data there are three (3) different Excel files (.xlsx) with datasets from the four (4) strain rates [2]. The excel files are titled, Dry untreated curly hair at 25 degrees, Wet untreated curly hair at 25 degrees and Wet untreated curly hair at 35 degrees. Each Excel file includes four worksheets. The worksheets are entitled, 10^0^, 10^−2^, 10^−3^ & 10^−4^ includes the strain stress data for ten (10) except for the 10^−4^ dry tests where the test numbers are six (6). The mechanical data composed of the Uniaxial stress- strain relationships was plotted at per Table 1 are shown on Fig. 1, Fig. 2, Fig. 3, Fig. 4 A-C for the different strain rates 10^0^.s^−1^,10^−2^. s^−1^, 10^−3^. s^−1^ & 10^−4^. s^−1^ (25°C dry condition, 25^°^C wet condition and 35^°^C wet condition), respectively.

## 4 Experimental Design, Materials and Methods

### 4.1 Specimen acquisition and preparation

The 116 specimens (N = 116) were collected from the African female individual in her early 30s to avoid the variation in mechanical properties influenced by age, gender, and ethnicity. The specimens were collected from the nape or occipital regions of the scalp as it is not too exposed to sunlight or other external environmental attacks. There was no cosmetic treatment done on the hair for the last six months, but only daily washing in water and drying was done on the hair prior to sample harvest. The specimens of 50 mm unstretched were harvested 5 mm from the scalp for morphology evaluation. The specimens without lags were 80mm in length then 25 mm lengths were removed from Both ends so that only 30 mm lengths were left for tensile testing. The specimens were washed and rinsed for ultimate cleaning and kept dry under an atmospheric state for 24hr to reach the equilibrium.

### 4.2 Morphology evaluation

Twenty per cent (20%) of the specimen was evaluated to confirm the curly profile before treatment and testing. The testing of the specimens was classified according to their curly pattern. The Wave Crest Assessment (WCA) method adapted from the previous study [3], [4] was used to identify the curl type. From the harvested 50 mm unstretched specimens, only a midspan length of 40 mm unstretched specimens were available for counting the number of crests and the 5 mm lengths on both ends were used to hold the specimens in place on the measuring ruler. Upon counting the crests, it was found that 80% of the sample had four crests while 20% had five crests which was then concluded that the specimens fell under type VI [5].

### 4.3 Mechanical tensile testing

The 20 mm single curly hair specimens (N=116) were tensile tested with the Ustretch 44N load cell as dry and wet conditions (25°C and 35°C) at the relative humidity 65% of tested under strain-rate 100.s-1,10-2. s-1 10-3. & 10-4s-1. The paper frame technique proposed by Adusumalli et al. [5] for the clamping of single hair fibers was then modified by using the A4 papers instead of the cardboard tabs used in the literature [6]. The lags on the specimen were removed by hand when gluing the specimen onto the paper. The fabric glue was used to glue the specimen onto the paper tabs. The 20 mm specimens were tested at room temperature, 24°C in dry conditions and in wet conditions at 25°C and 35°C. The specimens were stretched at 60% stretch magnitude.

As a concluding remark, it is worth mentioning that the datasets presented in this article contain information concerning real-life human curly hair fibers under different stress-strain in both dry and wet environmental conditions. This type of research is important for understanding the behaviour of curly hair fibers and can inform the development of more accurate 3D models for predicting their mechanical properties. By testing hair fibers in both dry and wet conditions, the study may have been able to capture the effects of moisture on the mechanical properties of the hair. The results of this study may help to identify the key factors that influence the mechanical properties of curly hair fibers and inform the development of new approaches to characterizing and modelling their behaviour.

## Limitations

The study's foremost limitation is its exclusive concentration on hair samples collected from a single individual of African descent. This restricted focus introduces inherent limitations pertaining to the extent to which the findings can be generalized beyond this specific individual and population group. It's crucial to recognize that human populations are incredibly diverse, and factors such as genetic variations, environmental influences, and cultural practices can significantly impact the characteristics and composition of hair. Therefore, while the study's results provide valuable insights for this specific context, their applicability to broader, more diverse populations may be limited.

## Ethics Statement

The study was granted ethical approval by the University of South Africa's Research Ethics Committee in the College of Science, Engineering and Technology [Ref: 2021/CSET/SOE/028].

## CRediT authorship contribution statement

**Lebogang Mathebela:** Conceptualization, Methodology, Data curation, Writing – original draft, Visualization, Investigation. **Rudzani Sigwadi:** Supervision. **Tetelo Lekwana:** Supervision. **Harry Ngwangwa:** Funding acquisition. **Thanyani Pandelani:** Funding acquisition, Writing – review & editing. **Fulufhelo Nemavhola:** Supervision, Funding acquisition, Writing – review & editing, Formal analysis.

## Data Availability

Dataset from the uniaxial tensile testing of human curly hair fibers under different conditions (Original data) (Mendeley Data).
